# Asymptomatic Carriage Rate, Multidrug Resistance Level, and Associated Risk Factors of Enterococcus in Clinical Samples among HIV-Positive Patients Attending at Debre Birhan Comprehensive Specialized Hospital, North Showa, Ethiopia

**DOI:** 10.1155/2023/7310856

**Published:** 2023-02-06

**Authors:** Mihret Tilahun, Alemu Gedefie, Zenawork Sahle

**Affiliations:** ^1^Department of Medical Laboratory Sciences, College of Medicine and Health Science, Wollo University, Dessie, Ethiopia; ^2^Department of Medical Laboratory Science, Debre Birhan Health Science College, North Showa, Ethiopia

## Abstract

**Background:**

Enterococci are facultative anaerobic, Gram-positive bacteria found in pairs and short chains that exist as normal microflora both human and animal. Enterococci have become a substantial source of nosocomial infections in immunocompromised patients, such as urinary tract infection (UTI), bacteremia, endocarditis, and wound infection. Earlier antibiotic therapy, length of hospital stays, and length of earlier vancomycin treatment, surgical wards, or intensive care units are all risk factors. Additionally, the presence of coinfections such as diabetes and renal failure and the presence of a urinary catheter were aggravated factors to develop infections. Data on the prevalence, antimicrobial susceptibility patterns, and associated factors of enterococcal infection among HIV-positive patients are scarce in Ethiopia.

**Objective:**

To determine the asymptomatic carriage rate, multidrug resistance pattern, and risk factors of enterococci in clinical samples among HIV-positive patients attending at Debre Birhan Comprehensive Specialized Hospital, North Showa, Ethiopia.

**Methods:**

A hospital-based cross-sectional study was conducted from May to August 2021, at Debre Birhan Comprehensive Specialized Hospital. To obtain sociodemographic data and possible associated factors of enterococcal infections, a pretested structured questionnaire was utilized. During the study period, clinical samples such as urine, blood, swabs, and other bodily fluids from participants sent to the bacteriology section for cultures were included. The study comprised a total of 384 HIV-positive patients. Enterococci were identified and confirmed using bile esculin azide agar (BEAA), Gram stain, catalase response, growth in broth containing 6.5% NaCl, and growth in BHI broth at 45°C. Data were entered and analyzed using SPSS version 25. *P* values < 0.05 with 95% confidence interval were considered statistically significant.

**Result:**

The overall asymptomatic carriage rate of enterococcal infection was 8.85% (34/384). Urinary tract infections were the most common, followed by wounds and blood. The vast majority of the isolate was found in urine, blood, and wound and fecal, 11 (32.4%), 6 (17.6%), and 5 (14.7%), respectively. Overall, 28 (82.35%) bacterial isolates were resistant to three and more than three antimicrobial agents. Duration of hospital associated with >48-hour hospital stays (AOR = 5.23, 95% C.I: 3.42-24.6), previous history of catheterization (AOR = 3.5, 95% C.I: 5.12-44.31), WHO clinical, stage IV (AOR = 1.65, 95% C.I: 1.23-3.61), andCD4 count < 350(AOR = 3.5, 95% C.I: 5.12-44.31) (*P* < 0.05). All were associated with higher level of enterococcal infection than their respective groups. *Conclusion and Recommendation*. Patients with UTIs, sepsis, and wound infection had a greater rate of enterococcal infection than the rest of the patients. Clinical samples in the research area yielded multidrug-resistant enterococci, including VRE. The presence of VRE suggests that multidrug-resistant Gram-positive bacteria have fewer antibiotic treatment options.

## 1. Introduction

Enterococci are facultative anaerobic Gram-positive bacteria typically arranged in pairs and short chains. The organisms are found in the oropharynx, vagina, urethra, and skin of healthy humans and animals as microflora [1]. They are catalase negative and grow at temperatures ranging from 10 to 45°C, pH 9.6, and 6.5% NaCl, as well as live for 30 minutes at 60°C. These organisms were hydrolyzing esculin in the presence of bile. Enterococci are commonly referred to as lactic acid producing bacteria which is the most common end product of glucose fermentation [2, 3]. Enterococci can appear as nonhemolytic, *α*-hemolytic, or, rarely, *β*-hemolytic colonies on enriched sheep blood agar that are enormous after 24 hours of incubation [4].

More than 67 species of *Enterococcus* are currently recognized; of these two species, *Enterococcus faecalis* and *Enterococcus faecium* are recognized as medically important [5]. Asymptomatic carriage rate and multidrug resistance are more common in community-acquired and immunocompromised individuals who are infected with human immune virus (HIV). Broad spectrum antibiotic therapy and frequent contact in the healthcare system can increase the risk of infection with multidrug-resistant (MDR) Enterococcus in patients with HIV having a weakened immune system. These people are more likely to get infections, including infections caused by bacteria that are resistant to antibiotics [6].

The rate of multidrug-resistant (MDR) enterococcal carriage is based on the immunological status and geographical region of the patient. Colonization of Enterococcus is usually asymptomatic, but it can last for months or years. It can spread MDR Enterococcus into the environment and to healthcare employees by shedding MDR Enterococcus [7, 8].

The spread of enterococcal infections has been linked to various risk factors, including earlier antibiotic treatment, coexisting infections, surgical procedures, catheterizations, duration of hospital stay, previous hospitalization, and chronic diseases such as cancer, HIV, and diabetics [9, 10]. In immunocompromised individuals, asymptomatic carriage rates of enterococci are the most common cause of infection. When the immunity level decreases, the spread and risk of enterococcal infections rises [11]. The increasing prevalence and spread of multidrug-resistant Enterococcus has resulted in patients suffering from serious illnesses; the evolution of antibiotic resistance in these organisms offers great challenges for clinicians [12]. The majority of *E. faecium* isolates are resistant to ampicillin and vancomycin, as well as penicillin, which are three of the most often used anti-Enterococcus drugs [13].

The prevalence of enterococci was described in the USA, Europe, Asia, Australia, and South America. The emergence of Enterococcus infection and their ability to resist multidrug therapy is also a hot issue in Africa, as studies in Nigeria, South Africa, and Ethiopia indicate Enterococcus prevalence ranging from 2.3% to 88.9% [14–17]. Understanding the asymptomatic carriage rate and associated risk factors, as well as studying the resistance pattern of Enterococcus species in immunocompromised individuals, is critical for reducing the infections. Therefore, this study is aimed at determining the asymptomatic carriage rate, multidrug resistance level, and associated risk factors of Enterococcus in clinical samples among HIV-positive patients attending at Debre Birhan Comprehensive Specialized Hospital, North Showa, Ethiopia.

## 2. Method and Materials

### 2.1. Study Design, Area, and Period

A facility based cross-sectional study was conducted from May to August 2021, at Debre Birhan Comprehensive Specialized Hospital, which is found in Debre Birhan town, North Shewa, Amhara Region, Ethiopia. Debre Birhan is located 130 km far from the capital city of the Addis Ababa, Ethiopia. According to the 2007 population and housing census of Ethiopia, the town has a total of 160,847 people. Pediatrics, emergency, surgery, medical gynecology, psychiatry, ophthalmology, antiretroviral therapy (ART), neonatal intensive care unit, microbiological laboratory, viral load, and other healthcare services are available. There are around 7000 HIV-positive clients at ART clinics of Debre Birhan Comprehensive Specialized Hospital. Debre Birhan is located at an elevation of 2840 meters above sea level, with an average yearly temperature of 10 to 16°C.

### 2.2. Inclusion and Exclusion Criteria

This study included HIV-positive patients who visited ART clinics. A total of 384 clinical samples such as fecal, ascetic fluid, blood, ear discharge, urine, pus, and CSF were collected. HIV patients who had received antibiotics other than cotrimoxazole less than two weeks ago were excluded.

### 2.3. Variables

The asymptomatic carriage rate and multidrug resistance pattern of Enterococcus were the dependent variables. In contrast, age, gender, residency, occupation, marital status, educational status, previous hospitalization history, history of previous catheterization, CD4, underlying chronic disease, hemoglobin level, previous history of antibiotic treatment except cotrimoxazole, patient setting.

### 2.4. Sample Size Determination and Sampling Technique

The sample size was calculated using a single population proportion formula with a 50% prevalence, a 5% margin of error, and a 95% confidence interval of 1.96 by using the sample size calculation formula below:

(1) $$\begin{array}{c} n=\frac{z^{2}p\left(1-p\right)}{d^{2}}, \end{array}$$

where *n* is the minimal sample size, *Za*/2 is the 95% of confidence interval significant value, *p* is the predicted frequency of enterococcal infection, and *d* is the margin of error. Using convenient sampling, a total of 384 HIV-positive people were included in this study.

### 2.5. Data and Specimen Collection

Sociodemographic, clinical, and risk factors were gathered through interviews guided by the use of a pretested structured questionnaire. Clinical samples were collected from each study participant in an aseptic manner. About 10 ml of blood from adults and 2 ml from pediatrics and infants were collected and dispensed aseptically into a blood culture vial containing 25 ml of Tryptic Soy Broth (FL Medical, Italy). Ten milliliters of freshly voided midstream urine was collected in a wide mouth, leak-proof, sterile plastic container and processed within two hours of collection. Approximately 5 ml of CSF and other body fluid samples was obtained aseptically and processed within one hour after collection. Using a sterile cotton tip applicator stick, wound swabs, pus, purulent exudates, wound discharge, and ear discharges were aseptically retrieved. All swabs were delivered within BHI broth, while the blood sample was transferred with the blood culture broth (HiMedia™). Each specimen was subsequently placed in a refrigerated box and delivered to the Debre Birhan comprehensive specialty hospital's Department of Microbiology Laboratory. Upon arrival at the laboratory, all specimens were inoculated immediately [18].

### 2.6. Isolation and Identification of Enterococci

To isolate the Enterococcus bacteria, specimens were inoculated on appropriate culture media. The blood culture bottles were incubated at 37°C for 24 hours each day for 7 days, looking for turbidity, hemolysis, gas generation, or color changes, all of which indicate microbial development. It was recorded as negative if the culture bottle did not exhibit any growth after 7 days. When apparent growth appeared, the bottle was aseptically opened, and a tiny amount of broth was removed with a sterile loop and subculture on bile esculin azide agar (BEAA) (Oxoid Ltd., UK). On BEAA media, urine samples were inoculated with calibrated loops and incubated for 24 hours at 37°C. Significant Enterococcus in urine were defined as those with >10^4^ colony forming units (CFU) per milliliter of urine and a black colored colony [19]. Other clinical samples were directly subcultured on BEAA and incubated at 37°C for 24 hours, with blackening media used to check for the formation of a very small colony. Enterococci were identified using colony features, Gram staining reaction, catalase, salt tolerance, and temperature tolerance test arabinose, sorbitol, and pyruvate. To distinguish commensal and pathogen species, the isolated organism was inoculated and can hydrolyze esculin in the presence of bile, releasing the product esculetin. Esculetin interacts with ferric citrate (in the medium) to generate a phenolic iron complex, which darkens to blackens the entire slant which indicate positive [19].

### 2.7. Antimicrobial Susceptibility Testing

The antimicrobial susceptibility testing of Enterococcus isolates was performed using the Kirby-Bauer disk diffusion technique based on the CLSI guideline 2021 [20]. About 3-5 colonies of bacteria were picked from a pure culture and transferred to a tube containing 5 ml sterile normal saline. The suspension was gently mixed to form a homogeneous solution, and the turbidity was adjusted to a McFarland 0.5 turbidity standard. The plates were streaked with a sterile cotton swab, and the excess suspension was removed by gently pushing and rotating the swab against the tube's interior wall surface. The swab was then used to evenly disseminate the germs across the Mueller Hinton agar surface (HiMedia). The inoculated plates were then left at room temperature to dry for 3-5 minutes, and a set of 6 antibiotic discs were applied on the MHA. The following antibiotics were tested based on CLSI recommendations and local prescription practice: erythromycin (15 *μ*g), chloramphenicol (30 *μ*g), tetracycline (30 *μ*g), ampicillin (10 *μ*g), ciprofloxacin (5 *μ*g), penicillin (10 IU), vancomycin (30 *μ*g), and amoxicillin-clavulanic acid (20/10 *μ*g). The standard antibiotics were purchased from Oxoid, Ltd., UK. The plates were then incubated for 16-18 hours at 37°C. The findings of the antimicrobial susceptibility pattern were interpreted using CLS criteria as sensitive, intermediate, and resistant [20].

### 2.8. Quality Assurance

Data quality was ensured by using standardized data collection materials. The questionnaire was pretested on 5% of the sample size in the nearby Debre Birhan Health Center before the actual study commenced to make sure whether the questionnaire is appropriate and understandable. Every day, the accuracy of data was checked by principal investigator. All laboratory analyses were carried out in accordance with standard operating procedures. All of the culture medium was made according to the manufacturer's instruction. Sterility of the culture media was done from the 5% the batch and incubated at 37°C for 24 hours. Reagents of Gram stain and biochemical testing media were checked by known positive standard strains *E. faecalis* ATCC 29212, *E. coli* ATCC 25922, *S. pyogenes* ATCC 19615, and *S. aureus* ATCC 25923, and negative controls and cotrimoxazole antibiotic drug were used.*Staphylococcus aureus* ATCC 25923 and *E. faecalis* ATCC 51299 were used as negative and positive controls for AST, respectively.

### 2.9. Statistical Analysis

Statistical Package for Social Sciences (SPSS) version 25.0 (IBM, USA) was used to enter and analyze the data, and descriptive statistics and binary and multivariate logistic regression were performed. The connection of each variable with the dependent variable was determined using bivariate analysis with maximum likelihood estimates of categorical variables. Additionally, factors with *P* < 0.25 in the bivariate analysis were subjected to multivariate logistic regression to identify the independent predictors of enterococcal infections. *P* value < 0.05 with 95% confidence interval was considered statistically significant.

## 3. Results

### 3.1. Sociodemographic and Clinical Characteristics of the Study Participants

A total of 384 study participants were involved with 100% response rate. Majority of them were 51.2% (199/384) males. The age ranges of study participants were from 1 day to70 years, with median and interquartile range of 20 and 2-36 years, respectively. About 105 (27.3%) of the study participants were the underage group of 16-30 years. The majority of the study participants 219 (76.10%) were inpatients, and 229 (59.6%) of the participants had a CD4 count > 350. Less than half of the respondents (150 (39.05%)) had low hemoglobin levels, and 142 (36.09%) had history of previous antibiotic treatment for <2 weeks. Most of the study participants (325 (84.63%)) did not have a previous history of hospitalization in the last three months, and 312 (81.25%) did not have a history of previous catheterization. Of the study participants, 121 (31.25%) had underlying chronic disease (Table 1).

### 3.2. Asymptomatic Carriage Rate of Enterococcal Infection

Among 384 study participants, 34 (8.85%) had an asymptomatic carriage rate for enterococcal infection. The asymptomatic carriage rate of the infection among inpatients and outpatients was 9.59% (*n* = 21) and 7.88% (*n* = 13), respectively. The proportion of asymptomatic carriage rate of Enterococcus species was higher among females (10.4%) than males. From a total of study participants, 15.8% were in the age group of <5 years with enterococcal infection, while 18.8% of the study participants with underlying chronic disease had asymptomatic carriage of Enterococcus (Table 1).

### 3.3. Distribution of Clinical Sample and Asymptomatic Carriage Rate

There were 80 urines, 41 pus, 53 blood, 16 ascetics, 10 ear discharge, 34 abdominal abscess, 80 CSF, 23 peritoneal fluid, 35 pleural fluid, 7 eye discharge, and 5 synovial fluid clinical samples analyzed. Most of the isolates were recovered from urine, blood, and pus (wound) and fecal, 11 (32.4%), 6 (17.6%), and 5 (14.7%), respectively (Table 2).

### 3.4. Antimicrobial Susceptibility Patterns

**Figure 1:**
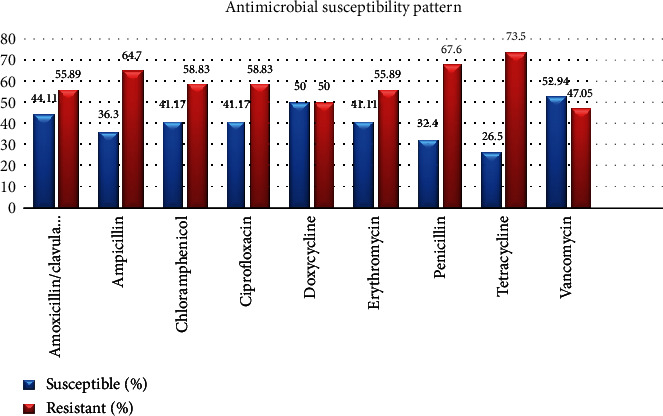
Antimicrobial susceptibility patterns of enterococci (*n* = 34) at the Debre Birhan comprehensive hospital, May to August, 2021.

The antimicrobial susceptibility patterns were performed against nine selected antibiotics for all the isolated of Enterococcus. The enterococcal isolates showed a higher level of drug resistance to tetracycline accounted 25 (75.5%) followed by penicillin 23 (73.5%) and ampicillin 22 (64.5%), while low level resistance rate to chloramphenicol 20 (48.83%), ciprofloxacin 20 (48.83%), and erythromycin 19 (55.89%) was reported for the rest of the antibacterial agents, respectively. Moreover, nearly half (16 (47.05%)) of the isolated Enterococcus were vancomycin resistant (Figure 1).

### 3.5. Multiple Drug Resistance Pattern of the Isolates

Overall, 34 (100%) enterococcal isolates were resistant to at least one antimicrobial agent, whereas 29 (85.3%) isolates were resistant to ≥2 antimicrobials. The overall multidrug resistances (MDR) were observed in 28 (82.35%) bacterial isolates which were resistant to three and more than three antimicrobial agents. Twenty-four (70.58%) isolates had developed resistance to five and more than five antimicrobials (Table 3).

### 3.6. Factors Associated with the Acquisition of Enterococcal Infections

In this study, all independent variables were subject to the bivariate analysis of risk factors for enterococcal infection. Factors like duration of hospital stays and previous hospitalizations, animal contact, who had cancer cases, previous history of catheterization, diabetics, patients who had UTI, WHO stage IV, and patients whose age group is 31-50 and CD4 count < 350 were statistically significant with COR (*P* value < 0.25) by binary analysis (Table 4). In the multivariate analysis, asymptomatic carriage rate of enterococcal infections was significantly associated with patients who have hospital stay ≥ 48 hours (AOR = 5.23, 95% C.I: 3.42-24.6), previous history of catheterization (AOR = 3.5, 95% C.I: 5.12-44.31), WHO clinical stage IV (AOR = 1.65, 5% C.I: 1.23-3.61), and CD4 count < 350 (AOR = 3.5, 95% C.I: 5.12-44.31) (*P* < 0.05 for all) (Table 5).

## 4. Discussion

The occurrence of Enterococcus, the evolution of drug-resistant Enterococcus, and the enterococcal colonization rate were increased with association to serious worldwide health concerns [21]. It might be due to different enterococcal isolates acquired drug resistant from diverse geographic sites; the epidemiology of Enterococcus is not well known.

The current study was conducted to determine asymptomatic carriage rate of enterococcal infection, evaluate the pattern of multidrug resistance, and identify possible risks associated with enterococcal infection among HIV-positive patients. The overall asymptomatic carriage rate of enterococci was 8.85% (34/384). This finding was consistent with the previous report of India (6.5%) [22] and Nigeria (5.9%) [23]. Similar findings were also reported in Ethiopia of Gondar (6.2%) [24] and Jimma (5.5%) [25]. However, it was lower than the previous report from Saudi Arabia (31.71%) [23], Tanzania (15.9%) [26], India (13.9%) [27], Saudi Arabia (14.1%) [28], Poland (30.7%) [29], the USA (45.2%) [30], and Dessie Ethiopia (37.3%) [31]. The current study was also higher than previous studies from different hospitals in Ethiopia such as Jimma (0.59%) [32], Felege Hiwot (0.64%) [33], University of Gondar Teaching Hospital (2.13%) [34], and Kenya (0.22%) [35]. The differences with the findings of this study may be attributed to the variation in study subjects, the extent of time, the method used for sampling, the different enterococcal detection methods used, and the use of traditional methods for identifying enterococci. The prior study respondents were hospitalized adults of pediatric age in an intensive care unit, which could explain the variance. Furthermore, the majority of the current study subjects were HIV-positive patients. The fact that immunocompromised patients were more susceptible to enterococcal infections may have contributed to the increased prevalence, as indicated by the current investigations.

In this study, the predominantly asymptomatic carriage rate of enterococcal species was isolated from urine specimens followed by blood and wound swabs and fecal, 11 (32.4%), 6 (17.6%), and 5 (14.7%), respectively. This was similar to previous reports conducted with previous report from India in which enterococcal UTI is 39.3% and wound infection is 15.3% [9] and other study done in Saudi Arabia in which bacteremia is 16% [23], whereas our finding was different from study done in Saudi Arabia in which UTI is 55% and wound infection is 9% [23] and another study in India in which UTI is 17.2%, bacteremia is 8.3%, and wound infection is 1.7% [36]. This demonstrated that enterococcal infection is the most common cause of urinary tract infection (UTI), bacteremia, and wound infection. The high frequency of enterococcal isolation from UTI cases is most probable due to the close proximity of the anal opening to the urethra where enterococci reside as commensals in the gastrointestinal tract. Uropathogens enter the bladder through the urethral opening after spreading over the perineum from the fecal flora. Furthermore, urinary catheterization played a role in some cases, contributing to greater enterococcal isolation from urine specimens.

Antibiotics resistance of bacteria is spread worldwide for the frequently used antibiotics [37]. In the present study, enterococci were more resistant to tetracycline (73.5%), penicillin (67.6%), ampicillin (64.7%), amoxicillin/clavulanic acid (55.89%), erythromycin (55.89%), ciprofloxacin (58.83%), chloramphenicol (58.83%), and doxycycline (50%). Similar findings were also reported in previous report from Gondar, Ethiopia, against penicillin (66.70%) [26] and report from India (52.6%) [38] and Jimma, Ethiopia (54.5%) [25]. However, the current study was higher than the previous studies to the following antibiotics: ampicillin 11.1%, ciprofloxacin 22.2%, chloramphenicol 22.2%, erythromycin 22.2%, and tetracycline 55.6% [34], in Iran [39], In India 42.3% [40], in Iran 69.7% [39]. However, the resistance pattern of erythromycin in current study was lower than was recorded in Gondar 71.6% [24], in West Amhara 94.0% [41], Gondar, Ethiopia 70.4% [42] as well as study done in Gondar, Ethiopia against ampicillin (100%) and erythromycin (87.5%) [42]. This might be due to the frequent use of irrational usage of the drug in the area. Additionally, in the case of our study, the participants were HIV patients who used different antibiotics along with HAART. This may contribute to microorganisms' drug resistance and lead to a high rate of drug resistance, including enterococci compared to other patients.

From the total 34 isolate enterococci, 10 (47.05%) were resistant to vancomycin. Similar findings were reported in a study conducted in Nigeria (42.9%) [43] and Gondar (41.5%) [24], but lower than the study conducted in Iraq (71.4%) [44] and Serbia (54.05%) [45]. The current result is also higher than a finding from Egypt (9%) [46]; it was higher than reports of other studies conducted among HIV patients in Ethiopia like Gondar (5.5%) [42], southern Ethiopia (7.5%) [47], Addis Ababa (6.7%) [48], Jimma (22.7%) [49], and west Iran (24%) [50] and reports of a nationwide study from Europe, Asia and Pacific, and Latin and North America (1% to 9.8%) [51]. This inconsistency might be due to the fact that the clinical sample in the case of Iraq was only urine and in the case of Serbia it was only blood. The emergence of VRE in several studies indicated the difference in the overall antibiotic resistance in the areas. Additionally, the progressive evolution of MDR strains, the difference in antibiotic selective pressure, local variation and changes in medicine prescription and usage behaviors, and socioeconomic level could all be factors.

The current study showed that enterococcal isolates had an overall multidrug resistance rate of 28 (82.35%). This finding was comparable with a study report from Spain (80%) [52], India (82%) [53], Jimma, Ethiopia (89.5%, 80.8%) [25, 49], southern Ethiopia (82.5%) [47], and Gondar (75%) [24]. However, it was considerably higher in comparison to other study findings from Dessie, Ethiopia (54.5%) [54], Germany (13.4%) [55], India (10%) [56], and Brazil (57%) [57]. The variations could be related to variances in the clinical sample used and the target study groups' ages. This was the study's most remarkable finding, and it is a concerning indication of multidrug resistance's expanding prevalence. The spread of drug-resistant strains, particularly in developing countries where drugs are readily available and inexpensive, could be a possible explanation for the rise in resistance patterns across enterococcal species. Furthermore, medical practitioners routinely prescribe these antimicrobial drugs as empirical therapy options, and self-prescribing is also common as a result of antibiotic overuse or misuse, as well as incorrect antibiotic prescription, which is a frequent practice in Ethiopia [58].

The multivariate analysis of the present study showed that patients having recent history of catheterization were significantly associated with pediatrics enterococcal infection (64.7%) compared to the relative groups (25.93%) and 8.5 time to acquire the infection with 8.5 (95% C.I: 5.12-44.31). This result was comparable with the study done in Spain (AOR = (95% C.I: 6.3 1.12–20.0)) [59]. Most enterococci are associated with medical device-mediated hospital-acquired infection [60]. The capacity of enterococci to acquire resistance genes and their intrinsic resistance to several commonly used antibiotics are the main reasons for their survival in hospital environments. Furthermore, the selective pressure exerted on sensitive strains as a result of antibiotic use plays a significant role [61]. Furthermore, most hospitalized patients are immunocompromised for a variety of reasons, including chronic diseases such as diabetes, cancer, and HIV, and enterococcal infections are common among immunocompromised patients [9]. Patients with a history of catheterization were also shown to have a significant correlation in our study.

The current study showed high prevalence of enterococci with patients having low CD4+ count < 350 (11.6%) compared to CD4+ count > 350 (7%). It was significantly associated with 3.6 times exposed with AOR = 3.6 (95% C.I: 2.1-9.76). A study conducted in a tertiary center in Northwest Ethiopia found a link between enterococcal infections and low CD4+ count [42] .

From 384 study participants, duration of hospital stay < 48 hrs is significantly associated with enterococcal bacterial infection with AOR = 5.23 (95% C.I: 3.42-24.6). The increased risk of insertion of various hospital devices, such as catheters, which is one of the main routes for transmitting nosocomial enterococcal infection in many health facilities, could be the cause of the increased prevalence in patients who stayed in the hospital for a longer period of time.

## 5. Limitation of This Study

The isolated *Enterococcus* as species level were not identified level, and molecular characterization was done due to limitation of resource.

## 6. Conclusion and Recommendations

In comparison to other studies, the prevalence of Enterococcus in HIV patients was rather low in our study. The asymptomatic carriage rate of the presence study was 8.85% the emergence of Enterococcus. Among them, 28 (82.35%) bacterial isolates were resistant to three and more than three antimicrobial agents. Enterococci were more resistant to tetracycline (73.5%), penicillin (67.6%), ampicillin (64.7%), amoxicillin/clavulanic acid (55.89%), erythromycin (55.89%), ciprofloxacin (58.83%), chloramphenicol (58.83%), and doxycycline (50%). The risk of infection rises when children have a history of chronic illnesses, hospitalizations, or invasive treatment procedures. Clinical samples in the research area revealed a high occurrence of multidrug-resistant enterococci, including VRE. Due to the high prevalence of MDR and VRE, antibiotic therapy choices for enterococcal infections are restricted. As a result, precautions should be taken to avoid nosocomial enterococcal infections and the spread of multidrug-resistant enterococci. For reasonable and appropriate antibiotic use, periodic monitoring of drug susceptibility patterns is also required. In addition, adequate antibiotic prescription and use, as well as large-scale species identification and genotypic research at the national level, are required.

## Figures and Tables

**Table 1:** Sociodemographic, clinical characteristics, and prevalence of enterococcal infection among patients (*n* = 384) at the Debre Birhan comprehensive hospital, May to August, 2021.

Variables	Enterococcal infection		Total
	Present no. (%)	Absent no. (%)	
Sex			
Female	19 (10.4)	166 (89.6)	185 (48.8)
Male	15 (7.4)	184 (92.6)	199 (51.2)
Age			
<5	3 (15.8)	11 (84.2)	19 (4.9)
5-15	5 (8.5)	54 (91.5)	59 (15.4)
16-30	10 (9.5)	95 (90.5)	105 (27.3)
31-50	9 (7.3)	114 (92.7)	123 (32)
>50	7 (8.97)	71 (91.03)	78 (20.3)
Residence			
Rural	20 (11.42)	155 (88.58)	175 (45.6)
Urban	14 (6.7)	195 (93.3)	209 (54.4)
Hemoglobin level			
Low	17 (11.33)	133 (88.67)	150 (39.06)
Normal	9 (4.86)	176 (95.14)	185 (41.17)
High	8 (16.32)	41 (83.68)	49 (12.76)
Previous history of antibiotic treatment except cotrimoxazole			
No	18 (9.74)	177 (90.26)	195 (50.78)
>2 weeks	9 (6.33)	133 (93.67)	142 (36.98)
<2 weeks	7 (14.89)	40 (85.11)	47 (12.24)
History of hospitalization in the last three months			
No	25 (8.12)	287 (91.88)	312 (81.25)
Yes	9 (12.5)	63 (87.5)	72 (18.75)
Underlying chronic disease			
No	12 (4.56)	251 (95.44)	263 (68.48)
Yes	22 (18.18)	99 (81.82)	121 (31.52)
History of previous catheterization			
No	18 (5.54)	307 (94.46)	325 (84.63)
Yes	16 (27.11)	43 (72.89)	59 (15.37)
Patient setting			
Outpatient	13 (7.88)	152 (92.12)	165 (23.90)
Inpatient	21 (9.59)	198 (90.41)	219 (76.10)
Education status			
Illiterate	14 (9.03)	141 (90.97)	155 (40.36)
Read and write	8 (8.25)	89 (92.75)	97 (25.26)
College and diploma	7 (8.33)	77 (82.67)	84 (21.88)
Degree and above	5 (10.41)	43 (89.59)	48 (12.5)
Total	**34 (8.85%)**	**350**	**384 (100.00)**

**Table 2:** Enterococcal distribution in different clinical samples (*n* = 34) at the Debre Birhan comprehensive hospital, May to August, 2021.

Type of sample	Frequency (%)
Fecal	5(14.7)
Blood	6 (17.6)
Ear discharge	2 (5.8)
Urine	11 (32.4)
Pus	5 (14.7)
Ascetic fluid	3 (8.8)
CSF	2 (5.8)
Total	**34 (100.00)**

**Table 3:** Multidrug-resistant profile of enterococcal isolates at the Debre Birhan comprehensive hospital, May to August, 2021.

Resistance rate	Combination of antibiotics	No. (%) of isolates tested
R2	TE, DOXY,	2
R3	TE, DOXY, CIP	2
R4	TE, E, DOXY, VAN	2
R5	TE, E, DOXY, CIP, CAF	3
R6	E, CIP, CAF, AMP, P, AMC	3
R7	TE, E, CIP, CAF, AMP, P, AMC	4
R8	TE, E, DOXY, CIP, CAF, AMP, P, AMC	4
R8	TE, E, DOXY, CAF, AMP, P, AMC, VAN	3
R9	TE, E, DOXY, CIP, CAF, AMP, P, AMC, VAN	7
Total MDR (*R* ≥ 3)		**28**

E = erythromycin; TE = tetracycline; AMP = ampicillin; P = penicillin; AMC = amoxicillin/clavulanic acid; CAF = chloramphenicol; CIP = ciprofloxacin; DOXY = doxycycline; VAN = vancomycin; R = resistance; R2-R9 = class of antibiotics resistance from 2 to 9, respectively; MDR = organisms resistant to ≥3 antibiotics.

**Table 4:** Bivariate analysis of associated factors for acquiring enterococcal infections at the Debre Birhan comprehensive hospital, May to August, 2021.

Characteristics	Total no. (%)	Enterococcal infection		COR (95% CI)	*P* value
		Presence no. (%)	Absence no. (%)		
Total	384 (100%)	34 (8.85%)	360 (91.15%)		
Sex					
Female	185 (48.8)	19 (10.4)	166 (89.6)	Ref	
Male	199 (51.2)	15 (7.4)	184 (92.6)	2.19 (0.59-3.98)	0.91
Age					
<5	19 (4.9)	3 (15.8)	11 (84.2)	0.93 (0.41-2.99)	0.55
5-15	59 (15.4)	5 (8.5)	54 (91.5)	0.88 (0.08-1.65)	0.52
16-30	105 (27.3)	10 (9.5)	95 (90.)5	0.36 (0.08-0.96)	0.51
31-50	123 (32)	9 (7.3)	114 (92.7)	1.23 (1.1-2.96)	0.09
>50	78 (20.3)	7 (8.97)	71 (91.03)	Ref	
Hospital stays (hours)					
<48	340	15 (4.6)	325 (95.4)	Ref	
48+	44	19 (43.2)	25 (56.8)	6 (4.32-25.6)	0.025
CD4 count^∗^					
<350	155 (40.4)	18 (11.6)	137 (88.4)	3.6 (2.1-9.76)	0.002
>350	229 (59.6)	16 (7.0)	213 (9.03)	Ref	
WHO stage^∗^					
I	165	4 (2.4)	161 (97.6)	Ref	
II	95	8 (8.4)	87 (91.6)	0.5 (0.00-25.1)	1.00
III	93	13 (14.0)	80 (86.0)	0.65 (0.00-3.71)	0.56
IV	31	9 (29.0)	22 (71)	1.85 (1.33-3.71)	0.006
History					
Antibiotics usage	84	7 (25.93)	77 (74.07)	Ref	
Animal contact	111	8 (9)	103 (90)	1.74 (0.73–4.58)	0.15
Cancer	13	2 (33.33)	11 (66.67)	7.80 (0.00–116.25)	0.18
Catheterization	47	9 (64.7)	38 (35.3)	8.5 (5.12-44.31)	0.001
Diabetics	39	2 (0.00)	37 (100)	4.5 (0.00-19.28)	1.00
UTI	90	6 (3.64)	84 (96.36)	0.53 (0.08-2.42)	0.55

**Table 5:** Multivariable analysis of enterococcal infections at the Debre Birhan comprehensive hospital, May to August, 2021.

Factors	AOR (95% C.I)	*P* value
Hospital stay > 48 hours	5.23 (3.42-24.6)	0.035
Hospital stay < 48 hours	Ref	
WHO stage^∗^		
I	Ref	
II	0.45 (0.00-23.1)	1.0
III	0.55 (0.00-2.71)	0.56
IV	1.65 (1.23-3.61)	0.035
Antibiotics usage	Ref	
Animal contact	1.11 (0.73–4.58)	0.35
Cancer	5.80 (0.00–16.25)	0.25
Catheterization	3.5 (5.12-44.31)	0.042
Diabetics	2.5 (1.00-15.28)	1.00
UTI	0.43 (0.05-1.42)	0.55
CD4 count^∗^		
<350	3.4 (2.1-9.76)	0.03
>350	Ref	

## Data Availability

Data auxiliary of this article is within the manuscript.

## Abbreviations

ATCC: American Type Cell Culture
BEAA: Bile esculin azide agar
BHI: Brain heart infusion
CDC: Centers for Disease Control and Prevention
CLSI: Clinical and Laboratory Standard Institute
CSF: Cerebrospinal fluid
DRH: Dessie Referral Hospital
HAIs: Healthcare-associated infections
HIV: Human immunodeficiency virus
IPC: Infection prevention and control
MDR: Multidrug resistance
MHA: Muller Hilton agar
SOPs: Standard operating procedures
SPSS: Statistical Package for Social Science
UTI: Urinary tract infection
VRE: Vancomycin-resistant Enterococcus.
